# DEVELOPING A COGNITIVE AND AFFECTIVE INTERVENTION FOR MILD COGNITIVE IMPAIRMENT FOR FOREIGN-BORN ARAB AMERICANS

**DOI:** 10.1093/geroni/igad104.0360

**Published:** 2023-12-21

**Authors:** Hala Darwish

**Affiliations:** University of Michigan, Ann Arbor, Michigan, United States

## Abstract

Cognitive and affective dysfunctions are challenging clinical characteristics of mild cognitive impairment (MCI) and Alzheimer’s disease and related dementias (ADRD), which may be more prevalent among foreign-born Arab Americans. One study reported that cognitive limitations were present among 9.7% of foreign-born Arab Americans, compared to 7.4% of US-born non-Hispanic whites, blacks, and Asians. This presentation will describe the design and development of culturally valid interventions targeting cognitive and affective dysfunction in foreign-born Arab Americans with MCI and their caregivers (NIH Stage 1B). Individuals with MCI and their caregivers will receive comprehensive cognitive rehabilitation and behavioral therapies. The cognitive performance will be assessed in Arabic using a culturally valid battery, including a verbal memory Arabic test (VMAT) we developed using a systematic item selection procedure (NIH Stage 1A). During Stage 1A, cultural and psychometric validity evidence for Arabic versions of the Symbol Digit Modalities Test (SDMT), Brief Visuospatial Memory Test-Revised (BVMT-R), and the newly developed VMAT was generated from a sample of 180 healthy Arabic-speaking individuals age 16-80 in Lebanon of whom 45 were 65-80 years, and 63 were retested after 2-3 weeks. Test-retest reliability and criterion-related validity were examined, and regression-based norms were derived. In Stage 1A, we also established the psychometric validity of Arabic measures of anxiety, depression, and fatigue (i.e., Hopkins symptoms checklist-25, modified fatigue impact scale) that will be used in the pilot randomized controlled trial (RCT)-stage 1B. A description of cultural adaptations and preliminary results will be discussed.

